# Medical Miscellanies

**Published:** 1818-06

**Authors:** 


### PART IV.

### MEDICAL MISCELLANIES.

Quicquid agunt Homines.

The unusual prevalence of contagious Fever in the City and Suburbs of Glasgow, has given rise to two pamphlets of very considerable ability, by two intelligent physicians there; viz. Dr. Graham, Professor of Botany; and Dr. Millar, Professor of Materia Medica in the Glasgow University.—We readily comply with the request of our Correspondent, to insert some interesting extracts from these publications; and we feel assured that our readers will have no cause to regret our having done so.

*The following Passages are from the Tract of Dr. Graham:*

" The general diffusion of an infectious disease throughout the country, must always happen whenever it establishes itself in a manufacturing town with such widely extended connections as Glasgow has. Every carrier leaving town, takes with him bales of infectious matter in the shape of cotton yarn, to be dropped at every village within fifty miles, and thence scattered indefinitely. At least, in one instance, the disease of which I am treating, has been traced from this to Edinburgh. In spring 1816, a beggar leaving this, carried it to a lodging-house in the Grassmarket, in which fourteen of the inhabitants were soon affected. The extinction of contagion, therefore, in such a situation as this, is not the concern of its own Police only, it may justly call the attention of the country in general; and if legislative interference could do any good, it would be fully warranted.

"Whatever difference of opinion there may be about the total disappearance of contagious fever in Glasgow, for some years previous to 1812, it is impossible for any man, who does not shut his eyes to the most direct evidence, to doubt that, at this moment, and for some time back, a much greater than usual number of fever cases exists; that they have been hitherto increasing in number, and that this increase has been most remarkable within the last six months. The number of fever patients which have been admitted into the infirmary, during the month of March, 1818, is 123, very considerably more than were admitted during any one year since its foundation, excepting 1799, when the number admitted was 128.

"The deaths among the males are very considerably greater than among the females. Of 601 patients under my own care, during the fourteen months I have stated, 288 were males, of whom 33 died; whereas, of 313 females, only 19 died, or rather more than 1 in 9 males, and about 1 in 16½ females. It is also a fact, acknowledged both in Ireland and every where in this country, that the mortality is much greater among the rich than the poor. Dr. Duncan, jun. is of opinion, that, in Edinburgh, the mortality, though greater among the rich, is less among the poor than usual. Do not these observations bear me out in the opinion first formed, from observation of the phenomena of the disease, that it is from the local inflammatory affections we have most reason to apprehend danger?

"All disease varies infinitely in its shape: certainly no complaint does so more constantly than fever; and, therefore, in none is there more necessity for leaving a discretionary power to the physician. There needs not, I think, a stronger proof of a man treating fever ill, than the circumstance of all his patients being treated alike.

"As I was fully saturated with all the prejudices against depletion, at my first acquaintance with these fevers, it is what I have seen that has compelled me, *nolens volens*, to adopt the sentiments which I now hold; and very fortunately for me, and most happily for the patients who have been under my care, I early met with some cases too decided in their nature to permit me to doubt. The first was a man with most intense head-ache, impetuous delirium, hard thrilling pulse, and the peculiar appearance of the eye, which I have attempted to describe.

"In these days I could not think of the lancet without trembling, but I ventured to order leeches to the forehead and temples. The relief was obvious, but the symptoms presently returned—the leeches were repeated—the symptoms again and again returned—the leeches were again and again applied; till, after much suffering and a great waste of time, the man permanently recovered.

"Imboldened by this success, I now ventured to direct blood to be taken from the arm of a man who had similar symptoms, and, in addition to the most painful restlessness produced by them, his whole body was constantly and greatly agitated by convulsive motions. When only six ounces of blood were withdrawn, this patient fell into a quiet sleep, never awoke till he was in all respects convalescent, and had not afterwards one unpleasant symptom. This was on the 21st day of the fever.

"It is needless to multiply examples to this effect; I shall only mention one other, the case of a man who lay in the same closet with this last. His symptoms were not so severe: they were as completely removed by venæsection, but they required blood to be drawn in much larger quantity, and they frequently returned, but he ultimately recovered by the repetition of the treatment.

"When stimuli are required, they must often be pushed in very great quantities. I have again and again been obliged to give wine almost *ad libitum;* and, not content with that, to exhibit alcohol, and ether, and camphor, in such doses as the patient could be made to swallow; and I have no doubt that, in some cases, he has owed his life to this treatment. External stimuli are also, in many cases, extremely useful; and, in doubtful ones, are safer far than those given internally. I greatly prefer sinapisms to blisters, when intended merely to rouse the system, on account of the facility with which they can be repeated, without the inconvenience of a filthy, and perhaps debilitating, discharge after each. When the feet are perfectly cold and benumbed, or when the patient complains much of pain in the toes, we must be on our guard against gangrene, and all its frightful consequences. All that I have found necessary to check this, is to rub the parts affected frequently in the course of the day, and for at least ten minutes at a time, with warm turpentine, taken into the palm of the hand; to wrap up the feet and legs afterwards in flannel, and to apply a heated brick.

"If any man wonders at the prevalence of continued fever among the lower classes in Glasgow, or at its spreading from their habitations, let him take the walk which I did to day, with Mr. Angus, one of the district Surgeons. Let him pick his steps among every species of disgusting filth, through a long alley, from four to five feet wide, flanked by houses five floors high, with here and there an opening for a pool of water, from which there is no drain, and in which all the nuisances of the neighbourhood are deposited, in endless succession, to float, and putrify, and waste away in noxious gases. Let him look, as he goes along, into the cellars which open into this lane, and he will probably find lodged, in alternate habitations, which are no way distinguished in their exterior, and very little by the furniture which is within them, pigs, and cows, and human beings, which can scarcely be recognised till brought to the light, or till the eyes of the visitant get accustomed to the smoke and gloom of the cellar in which they live. I have been to-day in several dens of this kind, where I did not see person's lying on the floor near me, till Mr. Angus, whom a previous visit had taught where to find them, inquired after their health. I was in one closet, measuring twelve feet by less than five, on the floor of which, he told me, six people had lain, affected with fever, within these few days, and where I saw the seventh inhabitant now confined. We found, in one lodging-house, fifteen feet long by nine feet from the front of the beds to the opposite wall, that fifteen people were sometimes accommodated; and when we expressed horror at the situation in which they were placed, the woman of the house, somewhat offended, and, I believe, a little alarmed lest we should cause some inquiry to be made by the Police, said, in support of the character of her establishment, that each family was provided with a bed, and that she very seldom had any body lying on the floor. I shall only mention one other instance of misery: In a lodging house, consisting of two rooms, separated by boards, the first thirteen feet by eleven, the other fifteen by eight, twenty-three of the lowest class of Irish were lately lodged. To-day there are fourteen, of whom two are confined with fever, three are convalescent, and one only has hitherto escaped. There are only three beds in this house (denominated, with that facetiousness which enables an Irishman to joke with his own misery, Flea Barracks), one of them in a press half way up the wall, the others wooden frames, on which are laid some shavings of wood, scantily covered with dirty rags. Most of the patients were lying on the floor. A man, two sons, and an adult daughter, were lying side by side on the floor of the first room, their bedding of the same materials with the others, and the boys being destitute of shirts. Could imagination feign a combination of circumstances more horribly conducive to disease and immorality!

"Mr. Angus tells me, that the father of this family is dead, that the Fever Committee have stepped from their usual rule, and sent one of the boys to the Relief Hospital; and that the only inhabitant of the house, who was well when I saw them, is now confined.

"Infectious fever was at one time fully more prevalent in Manchester than it has been in Glasgow; yet it has been eradicated there, and the judicious steps which have been persevered in, have kept that great population exempt from its ravages, at a time when it is visiting with more or less severity, almost every other crowded town in the empire, and even attacking with too much success, the healthy population of our country villages. The experience of London too is greatly in favour of the idea, that by proper measures we can keep fever under. Do not the whole quarantine laws proceed on the same supposition? and does not their success prove that the supposition is a correct one?

"Before the commencement of the present system for the suppression of contagious fever, the annual number affected with this disease in London was estimated at 40,000, and the deaths at 3,188. Now infectious fever is scarcely known at any of the London hospitals or dispensaries; and the number of cases at the House of Recovery is said to be seldom more than five or six.*

"An important step towards ventilation would be effected, if we could even open up the lanes in which the lower classes live. In Glasgow, the hovels which they inhabit are collected into dense masses of very great size between some of the larger streets. I believe it would greatly add to the healthiness of the place, if some improvements which I have heard talked of were effected, and straight and wide streets carried in different directions through these depositaries of wretchedness. It would not, I think, be easy to devise a more judicious charity, than the building of houses for the poor on an improved plan, and in a good situation. The avidity with which such houses are sought after, shows too, that it is a kind of benevolence which would cost very little money, if, indeed, it did not pay as a speculation.

"An attempt has been made to open the Riding School as a fever ward, and has met with such opposition from the inhabitants of the houses around, that I am sorry to find the idea is given up. The Committee have applied for many other buildings, but either the proprietors have refused to accommodate them, from a fear that the application of their property to such a purpose would prevent its letting again; or they have been resisted by the neighbouring families.

"The fear of infection from the neighbourhood of an hospital is altogether imaginary. The contagion cannot excite disease above a very few feet from the body infected, and it is very much to be regretted, that a mistaken prejudice of this kind should be permitted to obstruct so much real good, to scatter so much real misery, and, by increasing the extent of the disease, the quantity of the contagion, thereby to augment the risk of those very families who are at present arming themselves against an imaginary danger. There is not, I will venture to say, one case of fever contracted from the vicinity to the Royal Infirmary; and it is very well known, that the erection of a fever hospital in Gray's-Inn Lane, has had an effect the very reverse of fomenting the disease in that quarter. If, then, an Institution of this kind, has checked the disease in the middle of the smoke and filth of one of the most crowded districts in London, surely there is not the smallest chance of its diffusing it in a situation so free from every auxiliary to contagion as York Street."

*The following are from Dr. Millar's Publication:*

"During the last six years, that is, since 1812, the disease in question has been continually gaining ground among us, in Glasgow, and its neighbourhood. What is sufficiently remarkable, it appears before this period, for a short while, to have been almost

* This was the case until within the last twelve months, since which time, fever has been very prevalent in the metropolis, and the number of cases, at one time in the house of recovery, has occasionally amounted to upwards of sixty. Ed.

entirely extinct. On consulting the books of the Infirmary, a record of all others affording the most accurate data for learning the prevalence of any particular malady, at any particular time, among the poorer part of our population, I find that only sixteen cases of the distemper were admitted into the establishment during the whole currency of the year 1812. The most of these cases, too, did not occur during their usual season, the winter, but during the summer months. The same singular immunity from Typhus Fever, was, at this period, no less remarkable elsewhere. In London, Edinburgh, Manchester, Liverpool, and other large cities, its usual and favourite abodes, it was hardly to be seen.

"Since then it has been steadily creeping upwards, and since this æra, it will be found to have nearly doubled its numbers every successive twelvemonth. This will appear clearly from the following table of admissions into the Infirmary:

| Years. | Number of Admissions. |
|---|---|
| 1812 | 16 |
| 1813 | 35 |
| 1814 | 90 |
| 1815 | 230 |
| 1816 | 399 |
| 1817 | 714 |

"During the part of the present year that has already elapsed, the same regular increment is perceptible, the number in this quarter alone amounting to 303. March, the last month of the quarter, has been the worst of all, for I find the cases during this short period to be no less than 123. Supposing the same rate of increase to proceed during the remaining months of this year, and I see no reason why it should be less; on the contrary, it is probable to believe, that, instead of being diminished, it will be augmented; then the list of the sick in our infirmary, with continued fever alone, will, at the end of the twelvemonth, be exactly 1476. Now will this estimate, exorbitant as it may appear, be sufficient to convey to the public a proper idea of the extent of the contagion? To the above catalogue, large as it is, must also be added, numerous cases that for a good while past we have been obliged to turn away, however painful the necessity, from the doors of the Infirmary, absolutely on account of want of room and accommodation. In various others, besides, the sufferers possess neither means nor inclination for admission; and I have reason to believe there exists still a third class, who languish unassisted at home, and who have never been visited by any medical person whatsoever. In aggravation of all this calamity, it is further to be stated, that these instances of disease are not at present limited, as many of them were nine months ago, to particular places and districts; to certain alleys, lanes, and closes; they are now spread widely abroad, and in every direction. The whole persons thus infected, meanwhile, are continually generating more or less of typhus poison, and there have thus come to be engendered among us innumerable *foci* or *centres* of disease, each in unceasing activity and operation, by night as well as by day, and each diffusing its appropriate malignity round its little circle. The ramifications of contagion threaten thus to become indefinitely extended; and when we consider the multiplied links of connexion that bind together the upper and lower classes, as in the instance of servants, porters, washer-women, warehousemen, beggars, &c. &c. what house can be, at present, considered as secure against the entrance of the poison? Fortunately, the Infirmary has, hitherto, absorbed most of these foci of contagion; and in so doing, it has been the preservative of the town. Destitute of its aid, I know not by what miracle the atmosphere around us could have been so long exempted from almost universal pollution, or how the disorder could have been prevented, by this time, from extending it ravages through every class of the community.

"The house at Spring Gardens is calculated to receive 34 patients. The inadequacy of the new hospital for suppressing the contagion will appear the more manifest, if we consider some of the rules at first proposed for its management, and which could have been dictated only by the unfortunate narrowness of its accommodation. One of these was, that none but favourable cases were to be received; another, that all were to be excluded except those who resided within the municipal bounds of Glasgow. The first, if ever acted upon, I hold objectionable, in as much as it defeats one of the main purposes of the establishment, the facility of cleansing infected houses, by the prompt refuge it holds out to their diseased inmates. As for the second, it cannot be mentioned without a smile. Were the city surrounded by a wall of brass, the fever is an enemy that would make its way through that wall: Is it to be deterred by the barrier of a few Royalty stones dropped in its passage, though each be regularly numbered, and each have the letter R. as if it were a talismanic character, engraved upon it? New measures, therefore, or rather additional measures to what have been so happily begun, if we are to meet the present exigency, must be adopted.

"Instead of a Receiving House to hold 34, we must have one that will admit at least 100 to 120; or what is infinitely better, we ought to provide three or four different receptacles in different parts of the town, the united capacity of which shall be sufficient for the accommodation of the number I have mentioned.

"In the Gloucester Prison, one of the best regulated in England, it is a standing rule, that whoever enters must be previously subjected to a thorough purification by washing. Might not a similar precaution be useful, while the present epidemic lasts, in our own jail, Bridewell, &c. and indeed wherever a multitude of persons, especially if belonging to the lower orders, is collected together under the same roof? From our Bridewell, I have already had several cases of typhus under my care in the infirmary.

"Perhaps, as a supplement to what has been said about muriatic, oxy-muriatic, or nitrous acid gas, I ought not wholly to omit notice of some other, more popular, modes of prevention, such as the use of vinegar, either simple, camphorated, ammoniated or aromatic, often smelled to; the smoking, snuffing, chewing of tobacco, &c. All these I hold utterly useless, unless in so far as they may tend to promote a discharge from the mouth, a purpose for which the employment of tobacco, or any other masticatory, must, of course, be reckoned the most efficacious. There is, I think, some reason to believe, that particles of contagious matter floating in the atmosphere, may, now and then, be entangled in the saliva, so as to pass over with it into the stomach, and in that manner prove a source of fever. Those much occupied about the sick, therefore, should avoid swallowing their spittle; neither should they stand in a stream of air that blows over their beds. These two last are the only precautions I ever observe myself; that is, when I do not forget, as too often happens. In going my rounds, I have not unfrequently experienced some slight degree of squeamishness; but this almost immediately wears off on walking out into the open air.

"To me there appears complete evidence for the belief, that, every new portion of atmospheric fluid joined to the poison, robs it of more or less of its malignity, and that by constantly adding to it fresh quantities of air, we at last so dilute, or otherwise alter it, as entirely to deprive it of its fever-producing power. In every situation then, where this salutiferous plan can be practised, it is never to be neglected, but ought to be followed up with the utmost activity.

"I know, from unquestionable evidence, that much of our typhus contagion has proceeded from a certain description of low lodging-houses, destined for the accommodation of the poor, and of which the inmates, for some time back, have been chiefly indigent strangers (many from Ireland), who have resorted hither in quest of work. In these places, when an individual dies of fever, or is removed for cure to the infirmary, not the slightest effort is made to counteract the infection—on the contrary, a new inmate is received, who reposes in the same bed, is covered by the same bed-clothes, and is surrounded by the same furniture, so as constantly to breathe an atmosphere loaded with death and disease. As a natural result, he catches the distemper, augments the fomes in his turn, and finally makes way, either by removal to the infirmary, or dissolution, for a new inhabitant, who runs a similar course; and thus there is a constant succession of typhus patients from the same dwelling. To understand the enormity of this evil, it is only necessary to state its existence; it is one, however, to be rectified, not by medicine, but the Police."

The following passage is clever, and the allegation against his townsmen strongly put; yet, we *trust*—nay, we sincerely *believe*, that it is not altogether well founded, but that the ardour of composition, has hurried Dr. M. into a caricature rather than a real picture:

"An instance of hard-heartedness, and from the same cause fear of infection, has just now occurred. Elizabeth Howe, confined to bed with fever during several days, was, on the 12th of the present month, deliberately expelled her lodgings, by her landlord, into the street; the man, at the same time, telling her that he would not harbour his own sister, if in the same condition; and that, besides, she must be conscious she had no money to subsist upon during her illness. The poor woman, thus turned out, betook herself, for refuge, to different neighbours and acquaintances, who had the humanity to receive her, moving about from house to house. In the course of this perambulation she was, for 72 hours, without a bed to lie upon, but was always suffered to recline, or remain by the fire, in the night. Kindness, however, became exhausted, her neighbours and acquaintances grew tired of her, and she was again turned into the street. After this second expulsion, having lain down on a stair, she was discovered by one of the District Surgeons, and, through his humanity, conveyed to the Infirmary, where she at present remains under my care. This is a relation that requires no comment. I have often thought that were all the scenes of wretchedness occasioned by our epidemic, even only those which pass under the eyes of our District Surgeons, with others of the profession, who visit the mansions of the poor, collected into one narrative; they would form a curious, and certainly no inconsiderable chapter, in the history of human misery. What a noble act of charity would it be, to go the rounds of these desolate dwellings, cheering the minds of the inmates, and ministering to their wants; how congenial to that text of simple and incomparable pathos, preached from the Mount of Olives. "I was sick, and ye visited me;" how comfortable to the comment added by the same divine authority: "Inasmuch as ye have done it unto one of the least of these my brethren, ye have done it unto me!" But the character of "Marseilles good Bishop," I am afraid, is not very prevalent amongst us. Our piety has taken a new turn: it deals too much in generals. It delights only in objects at a distance, while it flies from those that are near. It must be pampered with something magnificent, what will figure in the telling, or may form an article in our new "Lettres Edifiantes et Curieuses." Nothing will serve it, but the high rank of Propagandist and Apostle. We glow with the desire of propagating the Gospel abroad; we are cold to its more practicable duties at home. The present miseries of the sick poor, in every alley, lane, and suburb of our city, are too gross and homely to attract regard; they want dignity or grace to allure the fastidiousness of our sympathy; they afford no outlet for the vanity of our godliness. We turn, with disgust or apathy, from crowds of tattered or dying wretches huddled together, a whole family often in a single bed, in hovels, some of them as airless as the Black Hole at Calcutta, destitute of every comfort and convenience, not unfrequently in want of food; and we console ourselves with the reflection, that our Bible Societies are flourishing, and that our Moravian Missions continue to prosper!"

### *Pulmonary Apoplexy.* Leroux.

Leroux relates the following case of what he denominates pulmonary apoplexy. M. Fortassin, one of the Editors of the *Dictionaire des Sciences Medicales*, was of middle stature, sanguine temperament, and very vigorous constitution. His neck was short, skin brown, and face a little florid. He never had experienced any illness in his life, except the small-pox. Although considering himself as in the enjoyment of good health, he was subject to hæmorrhoids, occasional dry cough, and sense of oppression about the chest. During the two days preceding his death, Fortassin had dined with good appetite, though moderately; supped on some dried fruit, and was in excellent spirits. At half past eleven o'clock he retired to rest, and stripped himself entirely. Being on watch with M Feller, over a man on whom a capital operation had been performed, his associate went into his chamber at half past three in the morning, and found him not only dead, but quite cold! He was lying on his face, his left hand across the chest, the right arm hanging out of bed; and his whole appearance indicated the great anguish which must have attended his dissolution. Some blood had issued from the mouth and nose.

*Post mortem examination.* The integuments of the face, neck, and chest, were injected with blood to such a degree as to be quite black, as though he had been strangled. On striking the thorax, the left side resounded, but not the right. The head being first opened, the brain, with all its investing membranes were perfectly healthy, and without any extravasation. The vessels and sinuses were not filled unusually. In the thorax, the heart and all the great vessels were sound and entire, but almost quite empty of blood, as in one who had died of hæmorrhage. The left side of the chest presented nothing remarkable. The lung of that cavity appeared sound, but on cutting into its substance, a sanguineous congestion was observed in its superior portion, and the bronchia of the same side contained some black blood. The *right* cavity of the chest was filled with black coagulated blood. The lung of this side was gorged as in the most intense pneumonia, and presented several deep rents. The substance of this lung was so completely denaturalized, (ellement dénaturée) and identified with the very compact clots of blood, that they could only be partially separated, and that with the greatest difficulty. On cutting this mass through in various directions with the scalpel, it was found impossible to distinguish the junction of the lung with the clots, or say where the one ceased and the other commenced. The bronchii of this side were filled with black blood, which was also found in the trachea, fauces and mouth.

Leroux observes, that there was no rupture of any arterial or venous vessel of any calibre, and that all the blood found in the right thoracic cavity had evidently escaped from the rents in the pleura pulmonalis of the gorged lung, and its parenchymatous structure. May we not say, adds the Professor, that the same thing took place in the lung here, which happens in the brain during those violent sanguineous apoplexies which instantaneously destroy life?

Leroux compares the above with the case of the late Professor Mahon, who struggled, however, a few days, during which he threw up a quantity of red and frothy bood. On opening the thorax, an extravasation of this fluid was found in the chest, but no rupture of any, even middle sized vessel.

### *Cutaneous Apoplexy.* RENAULDIN.

M. CONTINEAU relates the following case.

16th *Floreal, An.* 10. A soldier, 22 years of age, was brought into the hospital of Val de Grace, with the following symptoms: Considerable heat; full strong pulse; respiration quick; tongue loaded; abdomen tense and painful; belly costive. The skin presented a most singular appearance; it was universally scarlet in every part; and this colour seemed to depend on a red substance placed beneath the surface. The patient complained of the most exquisite pain over the whole body, but especially in the lumbar region, and uttered the most piercing cries on being moved on the bed. We could not learn that there had been any assignable cause for these phænomena. The red colour, the general pain, and the tenderness, had commenced simultaneously, and increased coequally. 17th. The above symptoms had all augmented in violence; the redness had extended to the sclerotic and transparent cornea; the pain had become insufferable; and the head was embarrassed, but without much delirium. In the morning, the pulse was pretty tranquil; but towards evening, it became small, hard, intermitting; and he died about midnight.

*Dissection.* Vessels of the brain very turgid; stomach and large intestines inflamed; the whole of the sub-cutaneous cellular tissue red, gorged uniformly with blood, but without any extravasation. The muscles contained a great quantity of blood, which issued forth on making incisions into their substance. In short, the whole capillary system of the body was completely injected.

M. Contaneau observes, that the disease was totally different from scarlatina, and that Lafon and Chagron had each met with a similar case, the one at Bourdeaux, the other at Brest.

These are interesting phænomena in hæmalogical researches, and may help to furnish data for a more accurate knowledge of the morbid states of the circulation than we yet possess. We shall ere long, return to this subject, which offers a wide and rich field for investigation. Ed.

### *Tetanus.*

We have been favoured with the minutes of an interesting case of Tetanus which occurred near Glasgow, in a healthy man, who had sustained a compound luxation of the thumb. The spasmodic symptoms supervened, with their customary violence, at the distance of ten days from the accident, and soon proved fatal, notwithstanding every means which the eminent practitioners in attendance had recourse to. In addition to the remedies ordinarily employed, blisters were applied repeatedly along the whole course of the spine, agreeably to the views entertained by Dr. Sanders, Dr. Esquirols, &c. as to the pathology of this disease, but without success. We regret to add that, owing to the prejudices of the patient's relatives, the body was not opened. The dissection would have been a highly interesting one.

### *Small-Pox.*

We are sorry to learn that several cases of small pox, *after vaccination*, have occurred in Edinburgh, in the course of the spring. In these cases the disease has generally been a mild one, though by no means uniformly so. The incipient fever preceding the eruption has always been considerable, and often severe; though, we believe, in no instance has any *secondary* fever taken place.

It not unfrequently was difficult to decide whether the eruption was small-pox, or chicken-pox of a severer form than ordinary. But it must be confessed, that amongst the cases that occurred, some were too well marked to be mistaken,—being the genuine small-pox.

We understand that Dr. Monro, Professor of Anatomy in the University of Edinburgh, has been carrying on a very minute and extensive enquiry into thereported failures of vaccination; and we hear that this distinguished gentleman means shortly to lay the result of his correspondence and enquiries before the public. We apprehend it will be an interesting document.

### *Injections of Cold Water, and Cold Water Half Baths, in Hæmorrhoidal Pains.*

Dr. Montegre, of Paris, has drawn the attention of the Faculty to the use of cold or chilled water injections, and half baths, in those distressing hæmorrhoidal pains which accompany the ejection of the fæces, where the patient is affected with piles. The following case is here given in illustration of this mode of treatment.

A man, 34 years of age, of an athletic, bilious, and sanguineous constitution, with very prominent veins, and born of hæmorrhoidinary parents, was himself subject to long and painful attacks of piles for the last five or six years. The last seizure continued three months, and he experienced no relief from any remedy, though a multitude were tried. Leeches only increased the inflammation and swelling of the tumours. Opium brought no relief. The pain was so great during each fæcal evacuation, that the patient was reduced to despair, and refused to take sustenance, lest he should add to his sufferings. In this situation he commenced the use of cold water injections and half baths, which, in a very few days so reduced the pain and external tumours, that he was able to push the latter within the sphincter ani. Five years have now elapsed without any hæmorrhoidinary attack.

*Report of the* CLIFTON DISPENSARY, *between the 29th of December*, 1816, *and the* 31*st of December*, 1817, *as transmitted by Dr.* DICKSON, F. R. S. and L. S. *Fellow of the Royal College of Physicians in Edinburgh, and one of the Physicians to the Clifton Dispensary.*

| Diseases. | No. | Diseases. | No. | Diseases. | No. |
|---|---|---|---|---|---|
| Anasarca | 4 | Erysipelas | 5 | Peritonitis Chronica | 3 |
| Aneurisma | 1 | Eruptiones sine Febre | 10 | Pertussis | 4 |
| Amenorhœa | 20 | Febris Contin. (Synochus) | 10 | Phthisis Pulmonalis | 16 |
| Apoplexia | 4 | —— Intermittens | 4 | Phthisis Incipiens | 14 |
| Aphtha | 4 | —— Remittens Infantum | 6 | Pneumonia | 79 |
| Ascites | 10 | Gastritis | 3 | —— Typhoides | 2 |
| Asthma | 6 | Hæmatemesis | 4 | Pleurodynia | 4 |
| Atrophia | 8 | Hæmoptysis | 5 | Pyrosis | 6 |
| Bulimia | 1 | Hepatitis Acuta | 12 | Rheumatismus Acutus | 21 |
| Carditis | 2 | —— Chronica | 16 | Rheumatismus Chronicus | 20 |
| Catarrhus | 5 | Hydrocephalus (Incipiens) | 13 | Rubeola | 3 |
| Cephalalgia | 5 | Hydrothorax | 3 | Scrofula | 10 |
| Cholera Morbus | 2 | Hypochondriasis | 4 | Syphilis | 1 |
| Colica | 4 | Hysteria | 6 | Tussis | 6 |
| Convulsio | 2 | Hysteria Hepatica | 8 | Typhus | 4 |
| Cordis Affectio | 5 | Icterus | 6 | Variola | 5 |
| Cynanche Tonsillaris | 6 | Mania | 3 | Varicella | 2 |
| —— Maligna | 2 | Mænorrhagia | 5 | Vermes | 15 |
| Dentitio | 3 | Nephritis | 7 | Vertigo | 5 |
| Diarrhœa | 26 | Obstipatio | 10 | Morbi Varii et Miscellanei | 31 |
| Dysenteria | 4 | Ophthalmia | 3 | | |
| Dyspepsia | 15 | Paralysis | 4 | Total | 564 |
| Enteritis | 8 | | | | |
| Enterodynia | 3 | | | | |
| Epilepsia | 16 | | | | |

*List of SURGICAL CASES for the Year* 1817.

| Diseases. | No. | Diseases. | No. | Diseases. | No. |
|---|---|---|---|---|---|
| Apostema | 15 | Hernia | 7 | Scabies | 11 |
| Abortio | 4 | Hernia Humoralis | 4 | Scrofula | 4 |
| Aneurisma | 1 | Hæmorrhois | 5 | Syphilis | 5 |
| Cancer | 2 | Inflammatio | 15 | Tic Douloureux | 1 |
| Caries | 4 | Luxatio | 2 | Tumor | 3 |
| Combustio | 5 | Obstetricatio | 4 | Ulcus | 59 |
| Concussio | 1 | Odontalgia | 3 | Vulnus | 12 |
| Contusio | 22 | Ophthalmia | 9 | Vaccinatio | 167 |
| Fractura | 3 | Porrigo | 2 | Miscellanei | 20 |
| Gonorrhœa | 11 | Procidentia Uteri | 3 | | |
| Hydarthrus | 3 | Psoriasis Diffusa | 7 | Total | 416 |
| Ischuria | 1 | | | | |

*GENERAL*

### *GENERAL STATEMENT.*

| | | |
|---|---|---|
| Total Number of Medical and Surgical Patients admitted for the year 1817 - - - - - - - - - - - - - | | 980 |
| Of which have been Discharged - - - | 915 | |
| Dead - - - - - - | 19 | |
| Remain under Cure - | 46 | —980 |
| Midwifery Cases - - - - - | | 87 |
| Grand Total - - | | 1067 |

### To Dr. JOHNSON.

My dear Sir,

Above I send you a List of the diseases of patients who have been under the care of the Physicians and Surgeons of the Clifton Dispensary during the last year, as drawn up by me from the books of that Institution.

As I was not connected with it until the latter part of the period which the Report embraces, I do not feel authorized to trouble you with many observations; but I hope on a future occasion to offer you some remarks on cases which have subsequently occurred, and upon some of the remedies that have been lately recommended particularly to the notice of practitioners.

The above Report comprises but few acute disorders belonging either to the Orders Phlegmasia or Exanthemata, with the exception of Pneumonia, which you will observe bears a great preponderance. Indeed, pulmonary complaints constitute above a fifth of the whole, and with Rheumatism, the next in order of frequency, must be considered as our grand climatorial diseases; as you have well illustrated in your very able work, lately published, on "The Influence of the Atmosphere of the British Isles."

From some cases of the chronic form of the latter disease, in which I have used the Belladonna and Stramonium with success, I am disposed to hope that these medicines will constitute a valuable addition to our practical resources, in some of the most troublesome Neuralgic complaints; and that those practitioners are entitled to our gratitude who add to the list of such remedies; for from Idiosyncrasy it frequently happens, that one medicine of this class may be used with the greatest advantage when others have failed; which again, in their turn, in cases apparently similar, had been previously exhibited with decided benefit.

A remarkable feature of the preceding Report deserves to be noticed: the fortunate infrequency of Fevers, especially those of a typhus character, which have proved so distressingly prevalent in various parts of the United Kingdom, and particularly in the sister island; affording a gratifying proof, that the sufferings and privations which have borne so heavily upon the poor every where, have been greatly ameliorated by the exertions of the charitable and humane in this populous parish. Upon the whole, although the number of indigent sick admitted by the Clifton Dispensary has been greater by 408 during the last year, the mortality has been less than in that which preceded it; and when the sum of disease that has been cured or alleviated during the above period is taken into consideration, it must afford reflections highly pleasing to the philanthropist, and to the supporters of this very useful Institution.

I remain, dear Sir, yours very faithfully,

Clifton, 1818. D. J. H. DICKSON.

---

ADVERTISEMENT.—Dr. JOHN B. DAVIS, Licentiate of the Royal College of Physicians of London; Physician of the Universal Dispensary for Children; Senior Physician of the London Dispensary; and Physician of the Surrey Dispensary, &c. &c. has commenced a Course of Lectures on that Branch of the Practice of Medicine which relates to the Diseases, Medicinal Management and Nursing of Children, at the Universal Dispensary for Children, No. 5, St. Andrew's Hill, Doctors' Commons.—In this Course, an extensive view will be taken of the peculiar circumstances of the Infant Economy during the different stages of that period, in order to prove the necessity of an appropriate plan of conduct in the Prevention and Treatment of Childrens' Diseases: the Doctrines of Disease will be amply illustrated by Clinical Observations and Cases drawn from the Records and Practice of the Dispensary; and Physiological Opinions of the Economy of Infants substantiated by facts, deduced from Comparative Anatomy in regard to the Economy of the Young of other Animals.

N. B. A Lecture will be delivered every Monday, at Twelve o'clock.

TERMS.—A single Course, £.4 : 4s.; a single Course and the Medical Practice for Three Months, £. 6 : 6s.; a single Course, and the Medical Practice for Six Months, £. 8 : 8s.

ADVERTISEMENT.—Dr. MERRIMAN, Physician-Accoucheur to the Middlesex Hospital, and Dr. LEY, Physician-Accoucheur to the Westminster Lying-In Hospital, will commence a Course of Lectures on the Theory and Practice of Midwifery, and the Diseases of Women and Children, on Monday the 1st of June, at half-past Ten o'clock. —Particulars may be learnt at Dr. Merriman's house, 26, Half-Moon Street, Piccadilly; at Dr. Ley's, South Audley Street, Grosvenor Square; and at the Middlesex Hospital, where the Lectures will be given.

M. La Baume has in the Press, "Observations on the Properties of the Air-Pump Vapour-Bath, pointing out their Efficacy in the Cure of Gout, Rheumatism, Palsy, &c.; with cursory Remarks on Factitious Airs, and on the improved State of Medical Electricity, in all its Branches, particularly in that of Galvanism, and their Efficacy in various Diseases."

END OF VOL. V.

---

☞ *The INDEX to this Volume will be given in our next Number.*

